# Anti-HER2 monoclonal antibodies intensify the susceptibility of human gastric cancer cells to etoposide by promoting apoptosis, but not autophagy

**DOI:** 10.1371/journal.pone.0255585

**Published:** 2021-08-26

**Authors:** Agnieszka Gornowicz, Wojciech Szymanowski, Robert Czarnomysy, Krzysztof Bielawski, Anna Bielawska

**Affiliations:** 1 Department of Biotechnology, Medical University of Bialystok, Bialystok, Poland; 2 Department of Synthesis and Technology of Drugs, Medical University of Bialystok, Bialystok, Poland; Columbia University, UNITED STATES

## Abstract

**Background:**

Gastric cancer (GC) is a multifactorial disease with high mortality. Anti-HER2 therapy is a promising strategy in GC treatment and trastuzumab was approved by FDA (Food and Drug Administration) as the first and the second line of treatment of the disease.

**Purpose:**

The aim of the study was to examine the effectiveness of a combination of etoposide with trastuzumab or pertuzumab in AGS gastric cancer cells and breast cancer cells such as MCF-7, MDA-MB-231 and HCC1954.

**Methods and findings:**

The cytotoxic effects of the tested compounds against gastric and breast cancer cells were checked by MTT (3-(4,5-dimethylthiazole-2-yl)-2,5-diphenyltetrazolium bromide) assay. The anti-proliferative potential was analyzed by the incorporation of [^3^H]-thymidine into DNA. Fluorescent microscopy and flow cytometry was used to demonstrate the effect of the compounds on apoptosis. The mitochondrial membrane potential, and the activity of caspase-8 and caspase-9 were assessed. Autophagosomes and autolysosomes formation was checked by flow cytometry. The concentrations of Beclin-1, LC3A and LC3B were performed using ELISA. The expression of LC3A/B was also determined. The results from our study proved that the combination of etoposide with anti-HER2 antibodies was not cytotoxic against breast cancer cells, whereas the combination of etoposide with anti-HER2 antibodies decreased viability and DNA biosynthesis in gastric cancer cells. The interaction of etoposide with pertuzumab or trastuzumab induced programmed cell death via extrinsic and intrinsic apoptotic pathways in AGS gastric cancer cells, but did not affect autophagy, where a decrease of Beclin-1, LC3A and LC3B was observed in comparison with the untreated control.

**Conclusions:**

The study demonstrated that etoposide (12.5 μM) with pertuzumab represent a promising strategy in gastric cancer treatment, but further *in vivo* examinations are also required.

## Introduction

Human epidermal growth factor receptor 2 (HER2) molecular pathway plays a significant role in the etiopathogenesis of many types of cancer and anti-HER2 therapy represents an important approach in targeted anticancer treatment [1]. HER2 receptors belong to the EGFR/HER family and are involved in the signaling, cell growth and differentiation of cells [2]. It was discovered that approximately 7–34% of patients with gastroesophageal cancer and 25–30% with breast cancer have overexpression or amplification of the HER2 gene [3]. Its abnormal expression was also found in other cancers like prostate cancer. In recent years a significant progress has been observed in the treatment of gastric cancer, but results are still unsatisfactory. Targeted agents in the group of HER2 inhibitors are still studied and different strategies are taken into account. Monoclonal antibodies (trastuzumab and pertuzumab) and tyrosine kinase inhibitors are commonly used in treatment of cancer with overexpressed HER2 [2,4]. Trastuzumab is a humanized monoclonal antibody that attaches to the extracellular binding domain of the HER2 receptor. The first indication for the use of this antibody was HER2+ metastatic breast cancer, but FDA approved it also in HER2 positive metastatic gastric cancer, where represents the first line of treatment [5]. Clinical trials, where the efficacy of antibody drug conjugates or tyrosine kinase inhibitors are still ongoing in HER2+ advanced gastric cancer [6]. The antibody drug conjugate (trastuzumab emtansine) showed promising tumor inhibitory effect in preclinical studies, but in one randomized trial, T-DM1 was not superior to chemotherapy in patients with HER2-positive advanced gastric cancer [7].

Pertuzumab belongs to the humanized antibodies and binds to the extracellular domain II of the HER2, thus preventing the formation of HER2/HER3 heterodimers. As a result, the HER2 intracellular domain does not phosphorylate and HER2 signaling activity is blocked. The safety and efficacy of pertuzumab in patients with breast cancer was documented in many clinical trials such as: CLEOPATRA, TRYPHAENA, APHINITY [6]. The combined use of two monoclonal antibodies: trastuzumab and pertuzumab enhanced the effect of inhibiting HER2 signaling activity, while at the same time increased the activity of immune mechanisms such as antibody-dependent cellular cytotoxicity and complement-dependent cytotoxicity (ADCC and CDC) [8–11].

Number of studies showed that combining monoclonal antibodies against specific targets with chemotherapeutic agents play a major role in treating patients with cancer. Etoposide represents a class of anticancer agents, which molecular mechanism of action is based on the inhibition of topoisomerase II and it is responsible for the accumulation of cells at G2/M phase [12]. Its antineoplastic activity as a single agent was proved in several malignancies including small cell lung cancer, lymphomas, ovarian and testicular cancer [13]. Etoposide is a component of two treatment regimens for patients with gastric cancer and it is used with doxorubicin and cisplatin or in combination with calcium folinate and 5-fluorouracil [14,15].

The aim of the study was to examine the effectiveness of the combination of etoposide with trastuzumab or pertuzumab in AGS gastric cancer cells with confirmed expression of HER2 [16] in comparison with monotherapy based on etoposide and untreated control. Additionally, the cytotoxic effect of etoposide alone and in combination with trastuzumab or peruzumab was checked in breast cancer cells such as MCF-7 (HER2-) and MDA-MB-231 (HER2-) and HCC1954 (HER2+). There are no studies, which include such a combination in treatment of gastric cancer. The study was performed to check whether anti-HER2 antibodies could intensify the susceptibility of gastric cancer cells to etoposide. The influence of monotherapy and combination of etoposide with anti-HER2 monoclonal antibodies on viability, DNA biosynthesis and molecular mechanism of induction of apoptosis was also demonstrated by a number of biochemical assays such as: determination of mitochondrial membrane potential, activity of caspases: -8 and -9. The effect of etoposide alone and in combination with pertuzumab or trastuzumab on autophagosomes and autolizosomes formation was conducted by flow cytometry. The concentrations of Beclin 1, LC3A and LC3B were assessed by ELISA to check the impact of the compounds on autophagy.

## Materials and methods

### Compounds

Etoposide was obtained from Sigma-Aldrich and the purity of the compound was >98%. Trastuzumab and pertuzumab were the products of Selleckchem. The purity of trastuzumab was 99.7% and the purity of pertuzumab was 99.17%.

### Cell culture

AGS-CRL-1739 human gastric cancer cells, MCF-7, MDA-MB-231 as well as HCC1954 breast cancer cells were obtained from American Type Culture Collection (ATCC). AGS, MCF-7 and MDA-MB-231 cells were cultured in Dulbecco’s Modified Eagle Medium (DMEM) and HCC1954 cells were maintained in RPMI-1640, which were supplemented with 10% fetal bovine serum (FBS) and 1% cocktail of penicillin and streptomycin. Cells were seeded in Costar flasks and grown in 5% CO_2_ at 37°C to reach about 90–95% of subconfluency. Then human gastric cancer cells were treated with 0.05% trypsin and 0.02% ethylenediaminetetraacetic acid (EDTA) in calcium-free phosphate buffered saline, counted in a hemocytometer and seeded in 6-well plates (Nunc) at 5 x 10^5^ cells/well in 2 mL of growth medium (DMEM). Cells that reached about 80% of confluency were used for further analysis.

### Cell viability assay

MCF-7, MDA-MB-231, HCC1954 and AGS-CRL-1739 cells were seeded in six-well plates and cultured as described above. MCF-7 and MDA-MB-231 cells were exposed for 24h to etoposide (50 μM) and its combination with trastuzumab or pertuzumab (10 μg/mL). HCC1954 and AGS cells were incubated for 24h with different doses (1 μM, 12.5 μM) of the tested compound (etoposide) as well as with its combination with trastuzumab or pertuzumab (trastuzumab + etoposide, pertuzumab + etoposide). MTT (3-(4,5-dimethylthiazole-2-yl)-2,5-diphenyltetrazolium bromide) was used as a reaction substrate and the absorbance of the converted dye in living cells was measured at a wavelength of 570 nm. Cancer cell viability in the presence of the analyzed agents was calculated as a percent of the control cells.

### [^3^H]thymidine incorporation assay

The effect of the studied compounds: etoposide as well as combination of etoposide (1 μM, 12.5 μM) with trastuzumab or pertuzumab (10 μg/mL) on cells proliferation was also tested. AGS-CRL-1739 cells were seeded in six-well plates and cultured as described above. Cells were treated with different concentrations of the tested compounds and 0.5 μCi of [^3^H]thymidine for 24 h at 37°C. The cells were harvested by trypsinization and washed several times in cold Phosphate-Buffered Saline—PBS (10 min/1.500 g) until the dpm in the washes were similar to the reagent control. Radioactivity was determined by liquid scintillation counting. [^3^H]thymidine uptake was expressed as dpm/well.

### Dual acridine orange/ethidium bromide fluorescent staining

AGS-CRL-1739 human gastric cancer cells were treated with the tested compounds for 24 h. The cell suspension (250 μl) was stained with 10 μl of the dye mixture (10 μM acridine orange and 10 μM ethidium bromide), which was prepared in PBS. Cells cultured in a drug-free medium were used as controls. The morphology of two hundred cells per sample was examined by fluorescent microscopy within 20 min. The results were analyzed with NIS-Elements software (Nikon Instruments Inc., Melville, NY, USA).

### Flow cytometry assessment of annexin V binding

The effect on the induction of apoptosis after 24h incubation with the compounds tested was determined by Becton Dickinson FACSCanto II flow cytometer FACSCanto II (Becton Dickinson Bioscences Systems, San Jose, CA, USA), assessing the loss of asymmetry of the phospholipids on the cell membrane. Cells were trypsinised, resuspended in DMEM and then in binding buffer. Next, they were stained with FITC Annexin V and propidium iodide (PI) for 15 min at room temperature in the dark according to the manufacturer’s instruction (FITC Annexin V Apoptosis Detection Kit II). Cells cultured in a drug-free medium were used as controls. Optimal parameter settings were found using a positive control (cells incubated with 3% formaldehyde in buffer during 30 min on ice). Forward scatter (FS) and side scatter (SC) signals were detected on a logarithmic scale histogram. FITC was detected in the FL1 channel (FL1 539; Threshold–value 52). The results were analyzed with FACSDiva software (Becton Dickinson Biosciences Systems, San Jose, CA, USA).

### Determination of mitochondrial membrane potential (MMP)

The lipophilic cationic probe 5,5’,6,6’-tetrachloro-1,1’,3,3’-tetraethylbenzimidazolcarbocyanine iodide (JC-1 Mitoscreen kit; BD Biosciences) was used to check the disruption of the mitochondrial membrane potential (MMP). Briefly, the unfixed cells were washed and resuspended in PBS supplemented with JC-1. AGS cells were then incubated for 15 min at room temperature in the dark, washed, and resuspended in PBS for immediate BD FACSCanto II flow cytometry analysis. The percentage of cells with disrupted MMP was calculated using FACSDiva software (both from BD Bioscences Systems, San Jose, CA,USA).

### Caspase 8 enzymatic activity assay

The FAM-FLICA Caspase 8 Kit (ImmunoChemistry Technologies, USA) was used to determine caspase 8 activity after the treatment of AGS-CRL-1739 cells with etoposide (1 μM, 12.5 μM) as well as a combination of etoposide (1 μM, 12.5 μM) with trastuzumab or pertuzumab (10 μg/mL) for 24h. After incubation, the cells were harvested and washed with cold buffer PBS. Then, 5 μl of diluted FLICA reagent and 2 μl of Hoechst 33342 were added to 93 μl of the cell suspension and mixed by pipetting. The gastric cancer cells were incubated for 60 min at 37°C. After that time, the cells were washed twice in 400 μl apoptosis wash buffer and centrifuged at 300 x g. After the last wash, the cells were resuspended in 100 μl apoptosis wash buffer and supplemented with 10 μg/ml PI. Analysis was performed using the BD FACSCanto II flow cytometer, and the obtained results of the study were analyzed with FACSDiva software (both from BD Biosciences Systems, San Jose, CA, USA).

### Caspase 9 enzymatic activity assay

The FAM-FLICA Caspase 9 Kit (ImmunoChemistry Technologies, USA) was used to analyze caspase 9 activity, measured according to the manufacturer’s instructions. The AGS-CRL-1739 cells were exposed to etoposide (1 μM, 12.5 μM) as well as a combination of etoposide (1 μM, 12.5 μM) with trastuzumab or pertuzumab (10 μg/mL) for 24 h. After that, the cells were harvested and washed with cold buffer PBS. FLICA reagent (5 μl) and Hoechst 33342 (2 μl) were added to the cell suspension (93 μl) and mixed by pipetting. The cells were incubated for 60 min at 37°C and then washed twice using a wash buffer and centrifuged at 300 x g. After the last wash, cells were resuspended in 100 μl apoptosis wash buffer and supplemented with 10 μg/ml PI. BD FACSCanto II flow cytometer was used to perform result analysis.

### Measuring the number of autophagosomes and autolysosomes by autophagy assay

To determine the effects of the tested compounds on the autophagy process of AGS gastric cancer cells, an autophagy assay was performed according to the manufacturer’s protocol. The probe was a cell-permeant aliphatic molecule that fluoresces brightly when inserted in the lipid membranes of autophagosomes and autolysosomes (Autophagy Assay, Red kit; ImmunoChemistry Technologies, Bloomington, MN, USA). In short, the unfixed cells were washed and then resuspended in PBS with the added autophagy probe, Red solution. Afterwards, the cells were incubated for 30 min at 37°C in the dark, washed, resuspended in cellular assay buffer and the provided fixative was added at a volume/volume ratio of 1:5. The samples were measured immediately after preparation by flow cytometry using the BD FACSCanto II system (BD Biosciences Systems). The percentage of cells with autophagy was calculated using FACSDiva software (BD Biosciences Systems). The equipment was calibrated with the BD Cytometer Setup and Tracking Beads (BD Biosciences, San Diego, CA, USA).

### Concentration of Beclin-1

A high sensitivity Human Beclin-1 SimpleStep ELISA (Abcam) kit was used to determine the concentrations of proteins in cell lysates from the AGS cell culture after 24 hours of incubation with the tested compounds, etoposide (1 μM, 12.5 μM) as well as combination of etoposide (1 μM, 12.5 μM) with trastuzumab or pertuzumab (10 μg/mL). Briefly, trypsinized cells were washed three times with cold PBS and centrifuged at 1000× *g* for 5 min at 4°C. The cells (1.5 × 10^6^) were suspended in lysis buffer for whole cell lysates. After centrifugation the supernatants were frozen immediately at −80°C. The standards, samples and the Antibody Coctail were added to the appropriate microtiter plate wells. After 1 hour of incubation at room temperature the microplate wells were aspirated and washed three times and then TMB development solution was added to each well. The enzyme-substrate reaction was terminated by the addition of stop solution and the color change was measured spectrophotometrically at a wavelength of 450 nm ± 2 nm. The antigen concentration in the samples was determined by comparing the O.D. of the samples to the standard curve.

### Concentration of LC3A and LC3B

High sensitivity assay kits (EIAab) were used to determine the concentrations of proteins in cell lysates from the AGS cell culture after 24 hours of incubation with the tested compounds. Briefly, trypsinized cells were washed three times with cold PBS and centrifuged at 1000× *g* for 5 min at 4°C. The cells (1.5 × 10^6^) were suspended in lysis buffer for whole cell lysates. After centrifugation the supernatants were frozen immediately at −80°C. The microtiter plate provided in this kit was pre-coated with an antibody specific to the analyzed antigen. The standards and samples were added to the appropriate microtiter plate wells. After 2 hours of incubation at 37°C, the plate was incubated with biotin-conjugated antibody for 1 hour at 37°C. Then, the microplate wells were aspirated and washed three times and then incubated with avidin conjugated to Horseradish Peroxidase (HRP). Next, a TMB substrate solution was added to each well. Those wells that contained the target antigen exhibited a change in color. The enzyme-substrate reaction was terminated by the addition of a sulfuric acid solution and the color change was measured spectrophotometrically at a wavelength of 450 nm ± 2 nm. The concentration of antigen in the samples was determined by comparing the O.D. of the samples to the standard curve.

### Antibody LC3A/B assay by flow cytometry

To check whether the tested compounds affect induction LC3A/B in AGS gastric cancer cells was using LC3A/B antibody conjugated to Alexa Fluor 488 (Cell Signaling Technology, Beverly, MA, USA), according to the manufacturer’s instructions. In brief, the centrifuged cells were suspended in 4% formaldehyde and incubated for 15 minutes at room temperature. The cells were washed by centrifugation with excess PBS. Afterwards, permeabilization was performed by adding ice-cold 90% methanol to the cells and incubating for 60 minutes in an ice bath. The cells were washed by centrifugation with excess PBS again. Then, they were suspended in 100 μl of diluted primary antibody, prepared in PBS at a 1:100 dilution, and incubated for 60 minutes at room temperature in the dark, washed and suspended in 300 μl PBS. The samples were measured immediately after preparation by flow cytometry using BD FACSCanto II. An analysis of the results was performed using FACSDiva software (both from BD Biosciences Systems). The equipment was calibrated with BD Cytometer Setup and Tracking Beads (BD Biosciences, San Diego, CA, USA).

### Statistical analysis

The results of the study are presented as mean ± standard deviation (SD) from three independent experiments. The statistical analysis was performed using GraphPad Prism Version 6.0 (San Diego, CA, USA). The ANOVA, and Tukey tests were used to demonstrate differences between the control cells and the cells exposed to varying concentrations of the tested compounds. A statistically significant difference was defined at p <0.05.

## Results

### The influence of etoposide alone and in combination with anti-HER2 monoclonal antibodies on the viability of MCF-7, MDA-MB-231 and HCC1954 breast cancer cells

**Fig 1:**
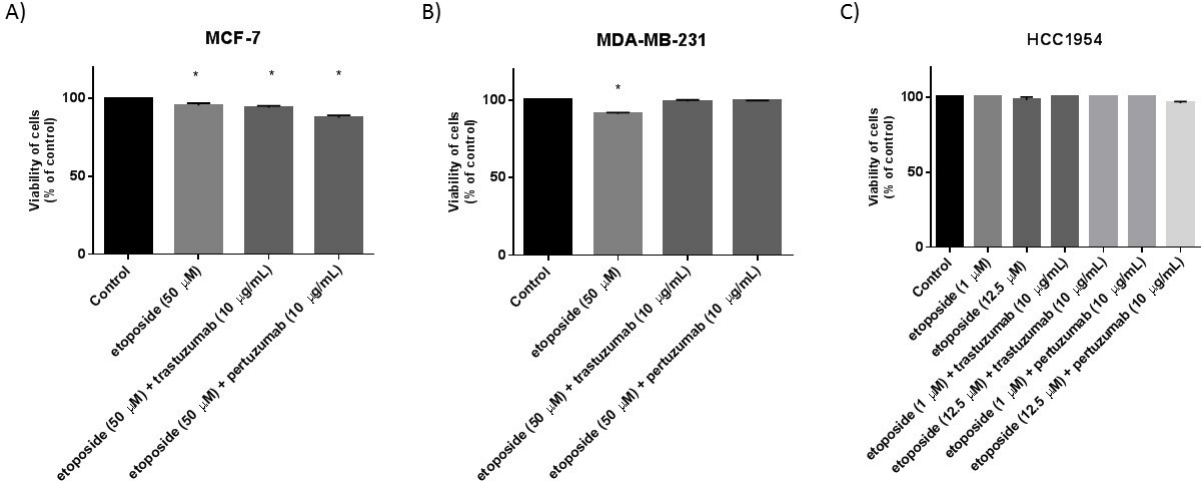
Viability of MCF-7 (A), MDA-MB-231 (B) and HCC1954 (C) breast cancer cells treated for 24h with etoposide alone and in combination with trastuzumab or pertuzumab (trastuzumab + etoposide, pertuzumab + etoposide). Mean ± SD from three independent experiments (n = 3) done in duplicate are presented. The ANOVA, and Tukey tests were used to demonstrate differences between the control cells and the cells exposed to varying concentrations of the tested compounds. *p<0.05 vs. control group.

The preliminary studies were performed to check the effect of etoposide and its combination with trastuzumab and pertuzumab on the viability of breast cancer cells. MCF-7 and MDA-MB-231, which are HER2 negative cell lines did not respond to the treatment (Fig 1A and 1B). In both breast cancer cell lines, a higher dose of etoposide was used (50 μM) and the results were not satisfactory. The similar effect was observed in HCC1954 breast cancer cells, which are HER2+. Etoposide alone as well as in combination with anti-HER2 antibodies did not decrease the viability of HCC1954 cells (Fig 1C).

### Etoposide with anti-HER2 monoclonal antibodies efficiently inhibits the viability and proliferation of human gastric cancer cells in comparison with the untreated control

**Fig 2:**
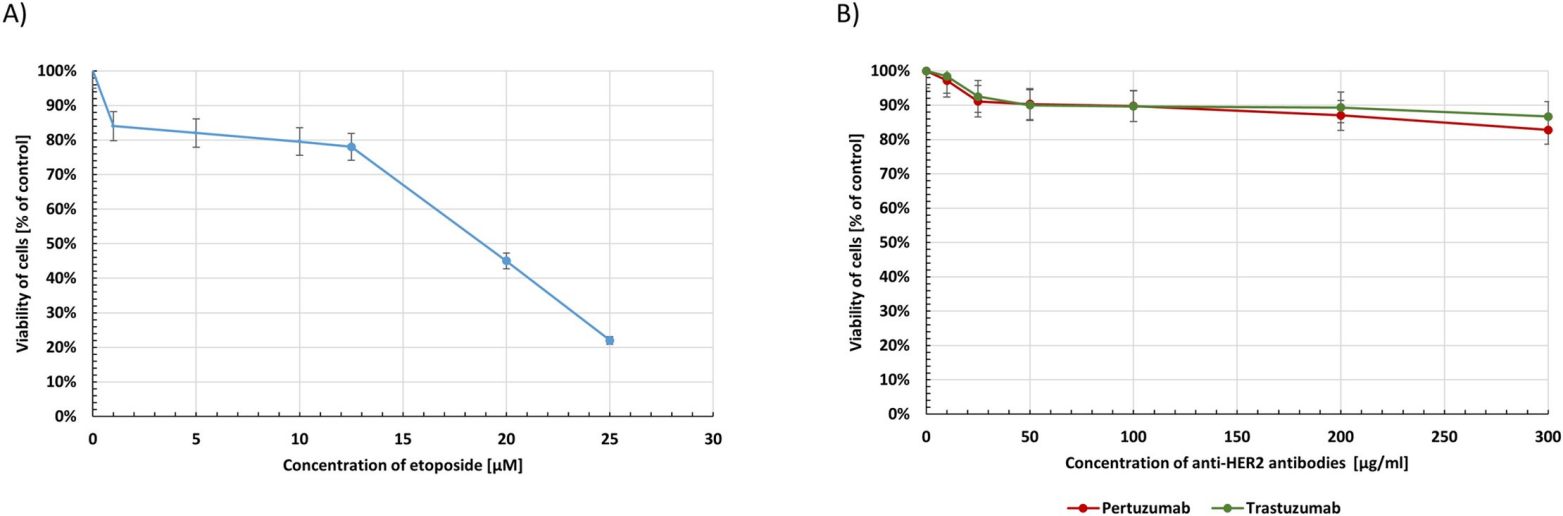
Viability of AGS gastric cancer cells treated for 24h with etoposide (A) and anti-HER2 antibodies (B) in different concentrations. Mean ± SD from three independent experiments (n = 3) done in duplicate are presented.

**Fig 3:**
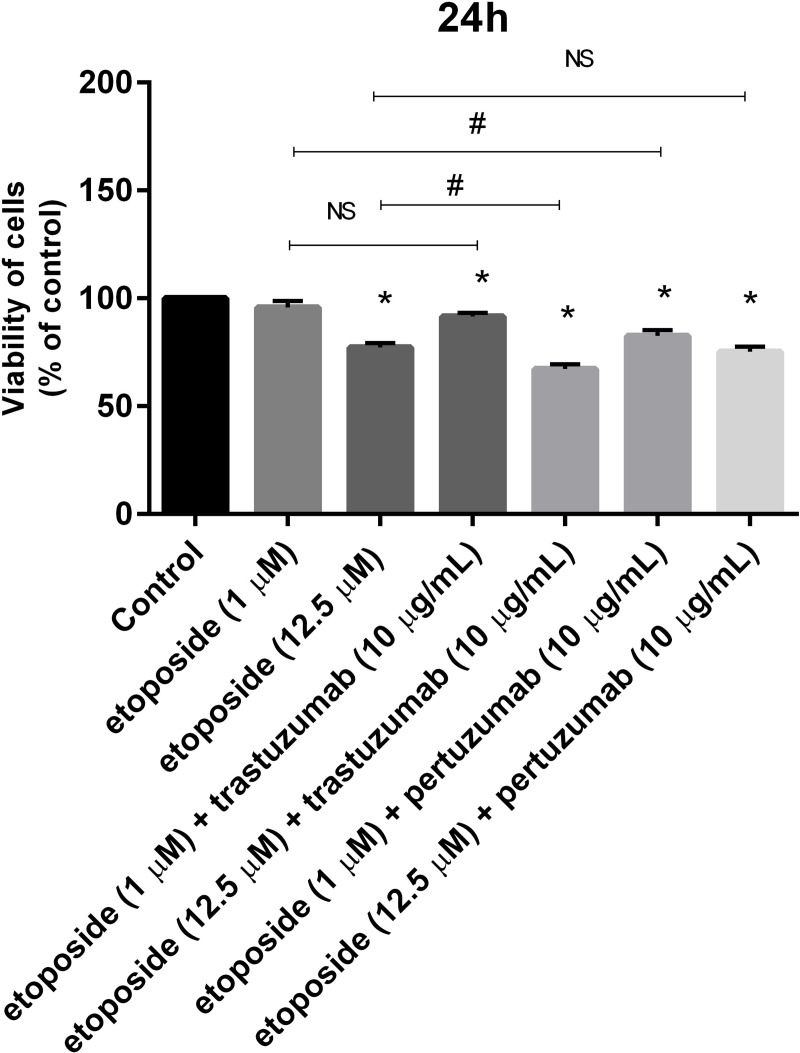
Viability of AGS gastric cancer cells treated for 24h with etoposide alone and its combination with trastuzumab or pertuzumab (trastuzumab + etoposide, pertuzumab + etoposide). Mean ± SD from three independent experiments (n = 3) done in duplicate are presented. The ANOVA, and Tukey tests were used to demonstrate differences between the control cells and the cells exposed to varying concentrations of the tested compounds. *p<0.05 vs. control group; #p<0.05. Ns, not significant.

The cytotoxic activity of etoposide, anti-HER2 antibodies and the combination of etoposide with trastuzumab or pertuzumab in human gastric cancer cells were checked after 24 hour incubation. The results are presented in Figs 2 and 3.

The etoposide was cytotoxic toward gastric cancer cells and the effect was associated with the dose of the chemotherapeutic agents. Its IC_50_ value was 19 μM (Fig 2A). The cytotoxic effect of pertuzumab and trastuzumab alone in broad spectrum of doses (10–300 μg/ml) was analyzed. Anti-HER2 antibodies were not so effective in decreasing the viability of AGS gastric cancer cells (Fig 2B). The addition of trastuzumab (10 μg/mL) or pertuzumab (10 μg/mL) to etoposide led to a decrease of cell viability. After the use of 1 μM concentration of etoposide with trastuzumab, we detected 82% of viable cells and 91% alive cells after the combination of etoposide (1 μM) with pertuzumab. Use of a higher dose of the chemotherapeutic agent (12.5 μM) enhanced the cytotoxic properties of such a combination. After 24-hour incubation of cells with etoposide and pertuzumab, the percent of viable cells was 75%, whereas the strongest anticancer potential was detected after the combination of etoposide with trastuzumab. In that case, we demonstrated 67.2% of viable cells (Fig 3).

**Fig 4:**
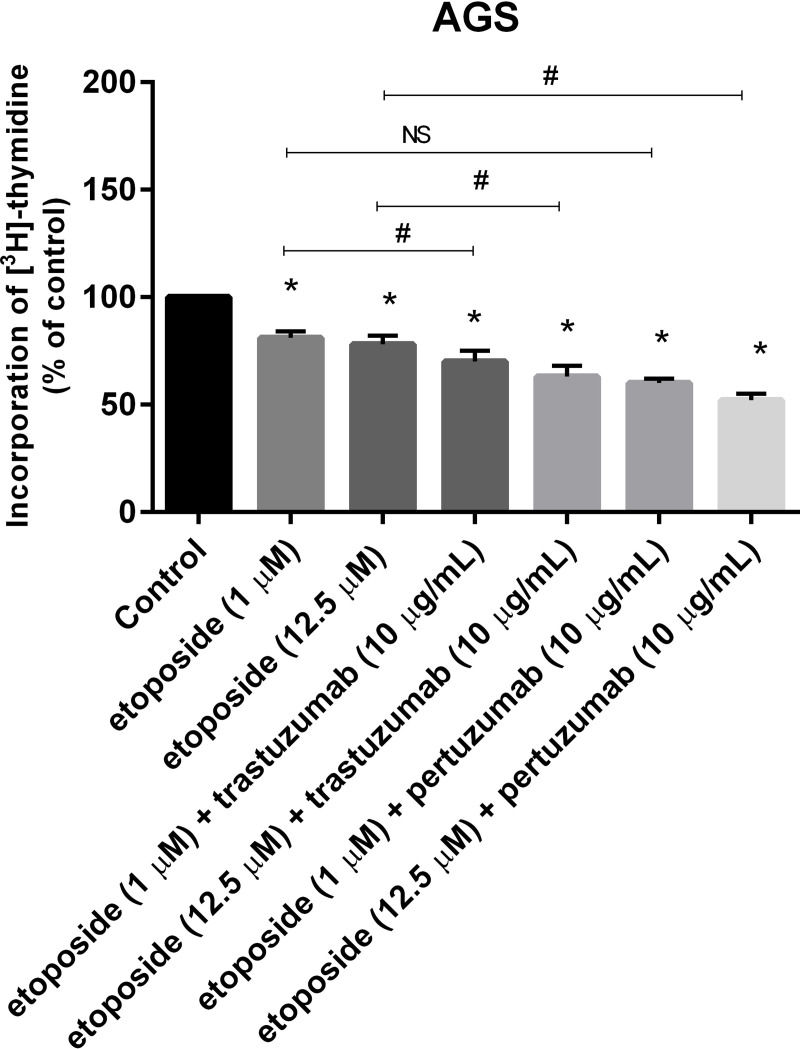
Antiproliferative effects of etoposide alone and its combination with trastuzumab or pertuzumab (trastuzumab + etoposide, pertuzumab + etoposide) in cultured AGS cells after 24h incubation, as measured by inhibition of [^3^H]-thymidine incorporation into DNA. Mean ± SD from three independent experiments (*n* = 3) done in duplicate are presented. The ANOVA, and Tukey tests were used to demonstrate differences between the control cells and the cells exposed to varying concentrations of the tested compounds. *p <0.05 vs. control group; #p<0.05. Ns, not significant.

Etoposide inhibited the biosynthesis of DNA in gastric cancer cells, but a stronger antiproliferative effect was indicated after 24-hour incubation with a chemotherapeutic agent and anti-HER2 monoclonal antibodies, especially with pertuzumab. After exposition of gastric cancer cells to etoposide in lower concentrations (1 μM), we detected the inhibition of the analyzed process up to 81%, whereas the addition of trastuzumab to etoposide resulted in the inhibition of the analyzed process up to 70% of the control. The most effective inhibitor of DNA biosynthesis was etoposide in combination with pertuzumab (60%). The higher dose of chemotherapeutic agent and monoclonal antibodies against HER2 more efficiently decreased the incorporation of [^3^H]–thymidine into the DNA than etoposide alone. The most significant decrease was revealed after 24-hour incubation with etoposide and pertuzumab, where the process was reduced to 52.6% of the control (#p<0.05) (Fig 4).

### Etoposide with anti-HER2 monoclonal antibodies induces apoptosis and decreases the mitochondrial membrane potential (MMP) in gastric cancer cells in comparison with the untreated control

**Fig 5:**
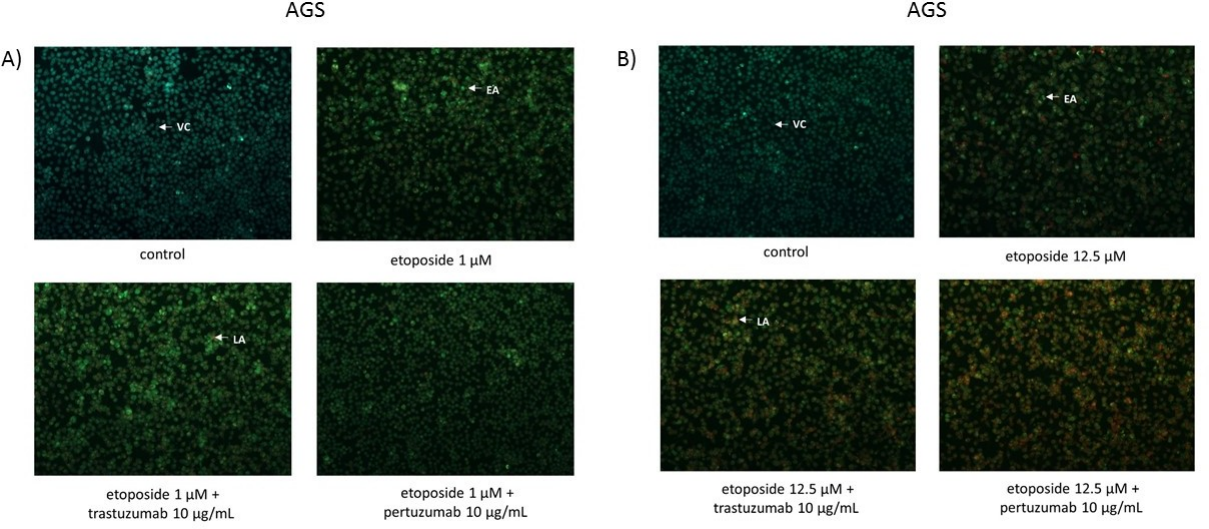
Induction of apoptosis in human AGS gastric cancer cells incubated for 24 h with etoposide (1 μM) and its combination with anti-HER2 antibodies (A) as well as with a higher dose of etoposide (12.5 μM) alone and in combination with anti-HER2 antibodies (B) was performed by a fluorescent microscopy after acridine orange and ethidium bromide staining (VC- viable cells, EA- early apoptosis, LA- late apoptosis).

**Fig 6:**
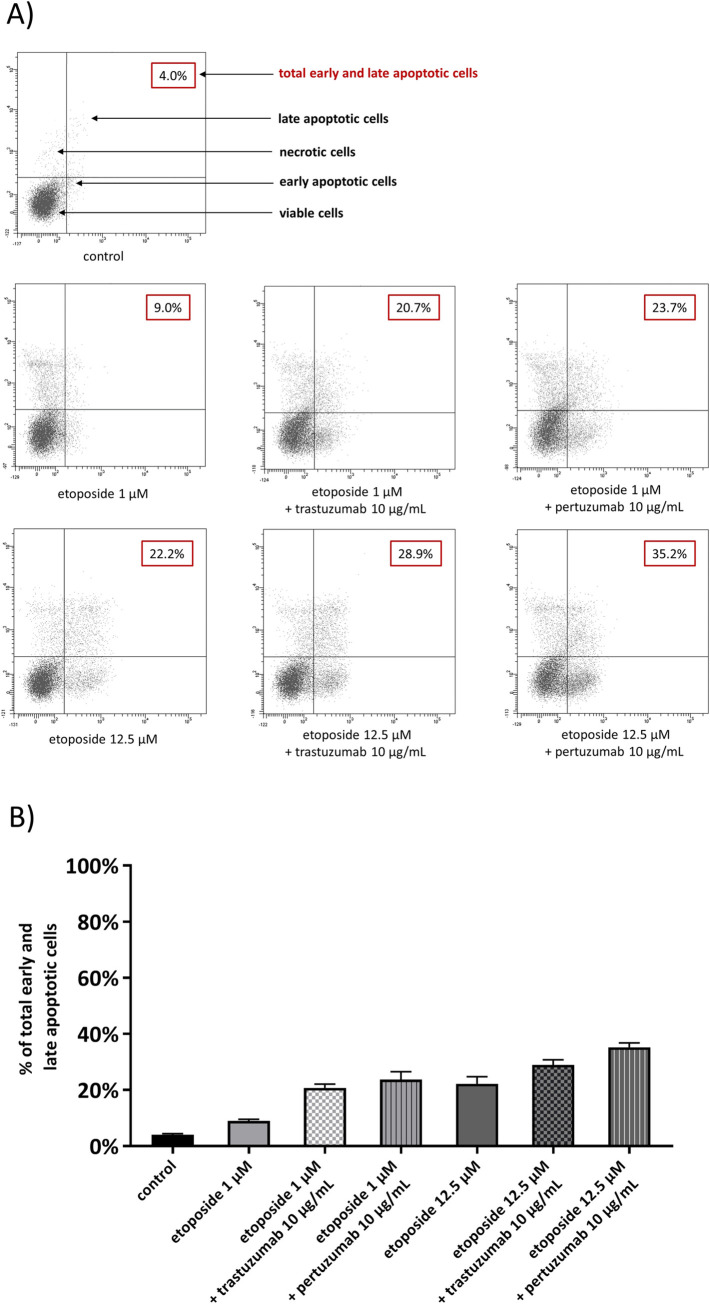
Flow cytometric analysis of AGS cells after 24 hour incubation with etoposide and its combination with anti-HER2 antibodies and subsequent staining with annexin V and propidium iodide (PI). Dots with annexin V^−^/PI^−^, annexin V^+^/PI^−^, annexin V^−^/PI^−^, and annexin V^+^/PI^+^ feature represent intact, early apoptotic, late apoptotic, and necrotic cells, respectively. Mean percentage values from three independent experiments (n  =  3) done in duplicate are presented.

**Fig 7:**
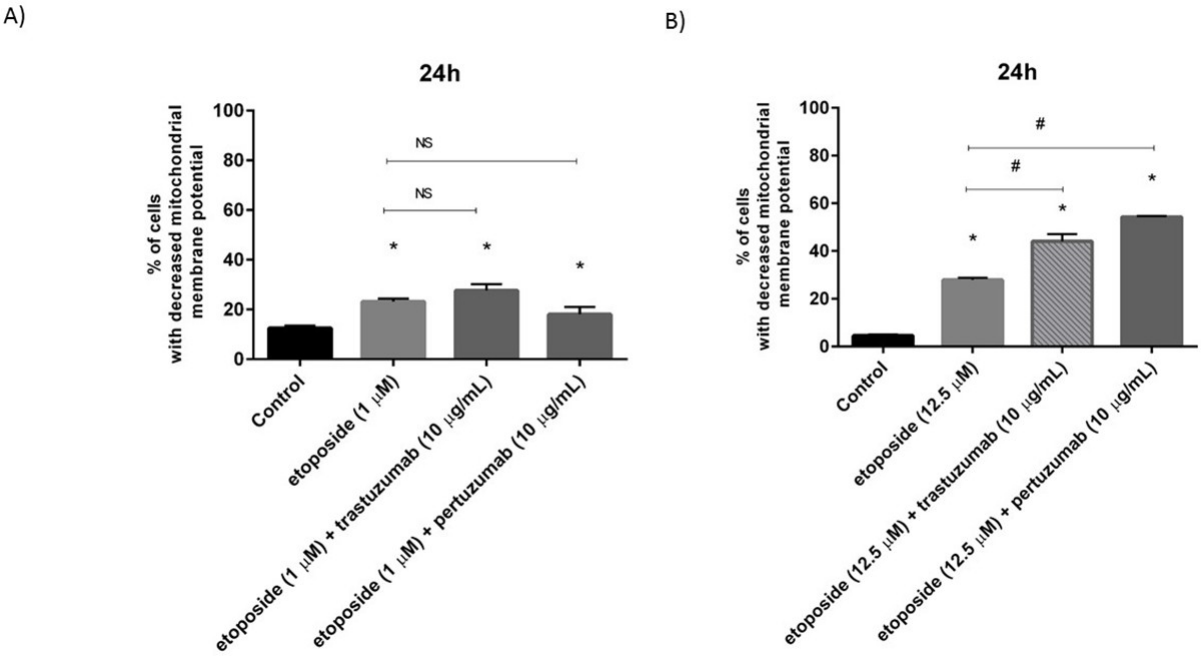
Decrease of mitochondrial membrane potential in cultured AGS cells after 24h incubation with etoposide (1 μM) and its combination with anti-HER2 antibodies (A) as well as with a higher dose of etoposide (12.5 μM) alone and in combination with anti-HER2 antibodies (B). The ANOVA, and Tukey tests were used to demonstrate differences between the control cells and the cells exposed to varying concentrations of the tested compounds. *P<0.05 vs. control group; #p<0.05. Ns, not significant.

After 24h incubation with the tested compounds, the induction of apoptosis was detected using fluorescent microscopy and flow cytometry. The results were presented in Figs 5 and 6. The staining of gastric cancer cells with acridine orange/ethidium bromide was performed to confirm the effect of the tested compounds on induction of apoptosis. The untreated cells with normal nuclear structure were identified as green fluorescence. The early stage of apoptosis was displayed as bright green fluorescence, whereas the late apoptosis was identified by reddish-orange color. Our observation led to the conclusion, that higher dose of etoposide with pertuzumab or trastuzumab strongly initiated the analyzed process. We detected higher number of early and late apoptotic cells in comparison with the untreated control (Fig 5B). The highest percentage of early and late apoptotic cells (35.2%) was demonstrated after 24 hour exposition to pertuzumab with etoposide (12.5 μM) (Fig 6). The decrease of mitochondrial membrane potential was checked to support the results evaluated by a fluorescent microscopy and flow cytometry. After treatment with the compound in a dose of 1 μM alone and in combination with anti-HER2 monoclonal antibodies, we demonstrated a higher decrease in the mitochondrial membrane potential in comparison with the control. The strongest effect was observed after 24-hour incubation with etoposide and trastuzumab, where we detected 27.6% of cells with decreased MMP. We also demonstrated 23.2% of cells with decreased MMP after treatment with etoposide and 18% of cells with lower MMP after treatment with etoposide and pertuzumab (Fig 7A). All values were statistically significant compared with the control (p<0.05). Increasing the dose of etoposide to 12.5 μM led to a significant decrease of MMP in the analyzed gastric cancer cells. The combination of etoposide with pertuzumab diminished the mitochondrial membrane potential in 54.3% of the analyzed population of cells, whereas use of etoposide with trastuzumab led to the inhibition of MMP in 44% of the population of gastric cancer cells. The weakest effect was observed after 24-hour incubation with etoposide alone, where 27.9% of cells had lower MMP (Fig 7B).

### Combination of etoposide with anti-HER2 monoclonal antibodies increases the activity of initiator caspases in gastric cancer cells

**Fig 8:**
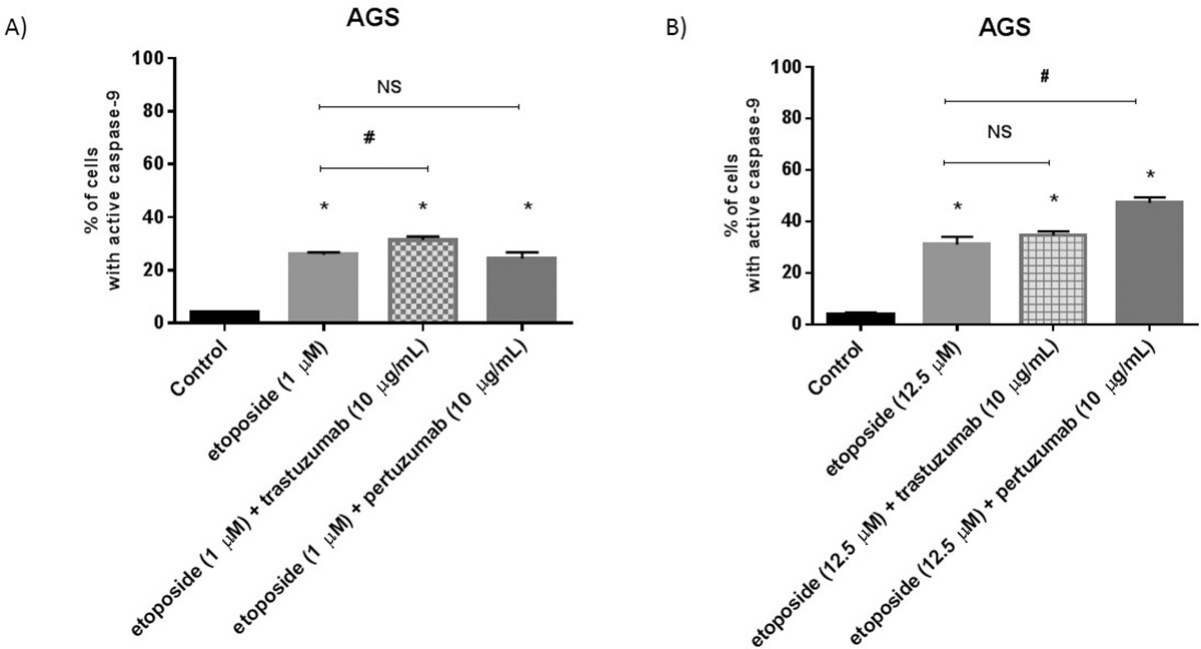
Activity of caspase-9 in AGS cells after 24h incubation with etoposide (1 μM) and its combination with anti-HER2 antibodies (A) as well as with a higher dose of etoposide (12.5 μM) alone and in combination with anti-HER2 antibodies (B). The ANOVA, and Tukey tests were used to demonstrate differences between the control cells and the cells exposed to varying concentrations of the tested compounds. *p<0.05 vs. control group; #p<0.05. Ns, not significant.

**Fig 9:**
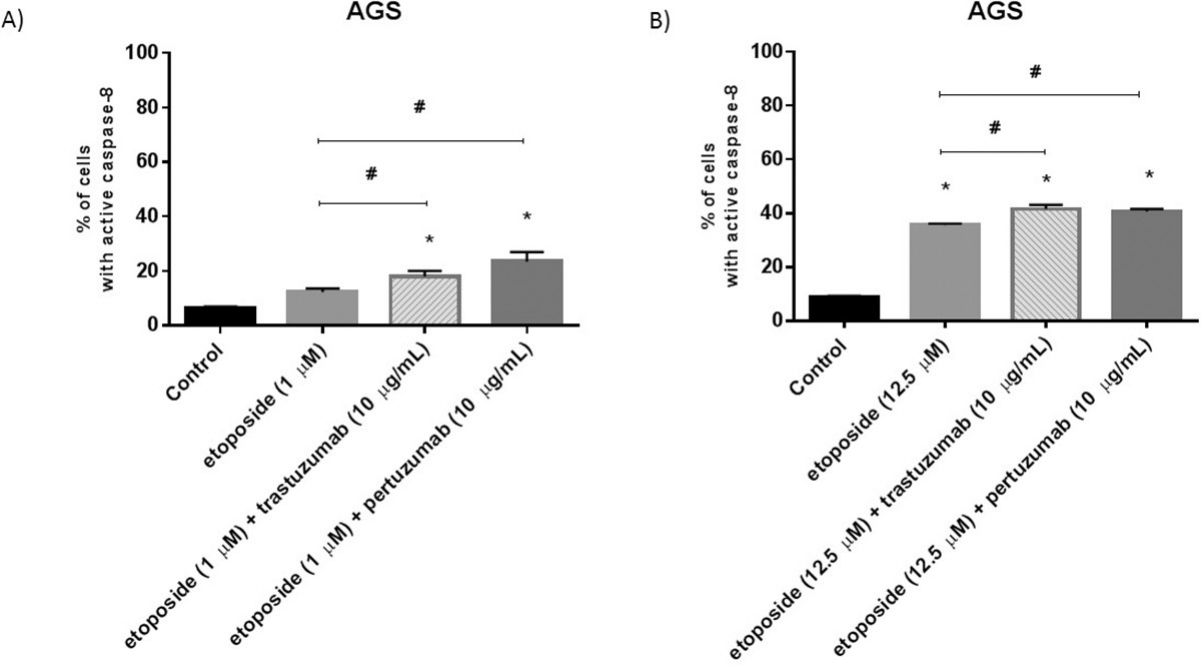
Activity of caspase-8 in AGS cells after 24h incubation with etoposide (1 μM) and its combination with anti-HER2 antibodies (A) as well as with a higher dose of etoposide (12.5 μM) alone and in combination with anti-HER2 antibodies (B). The ANOVA, and Tukey tests were used to demonstrate differences between the control cells and the cells exposed to varying concentrations of the tested compounds. *p<0.05 vs. control group.

It was well documented that cancer cells avoid programmed cell death [17]. We checked the effect of our tested monotherapy and combination of compounds on the activity of initiator caspase-8, whose role in the external apoptotic pathway is uncontested, as well as caspase-9 responsible for the activation of the mitochondrial apoptotic pathway. The administration of cells to etoposide confirmed its proapoptotic potential. We proved that both caspases were activated and the effect was dependent on the dose of the chemotherapeutic agent. We showed that 25.9% of cells with active caspase-9 and 12.4% of cells with active caspase-8 after treatment with 1 μM concentration of etoposide (Figs 8A and 9A). The higher dose of etoposide (12.5 μM) affected the activation of both caspases more efficiently than in control. We observed 31% of cells with active caspase-9 and 35.6% of the analyzed population with active caspase-8 (Figs 8B and 9B).

The exposition of cells to the combination of a higher dose of etoposide with anti-HER2 monoclonal antibodies especially enhanced the activity of caspase-8 in comparison with monotherapy (#p<0.05). After 24h incubation with etoposide and trastuzumab (12.5 μM + 10 μg/ml), we observed 41.6% of cells with active caspase-8 and 34.6% cells with active caspase-9, whereas the exposition to etoposide with pertuzumab (12.5 μM + 10 μg/ml) also strongly resulted in the activation of caspase-8 (40.8%) and caspase-9 (47.2%) (Figs 8B and 9B). All values were statistically significant in comparison with the untreated control (*p*<0.05).

### Combination of etoposide with anti-HER2 monoclonal antibodies does not induce autophagy in gastric cancer cells

**Fig 10:**
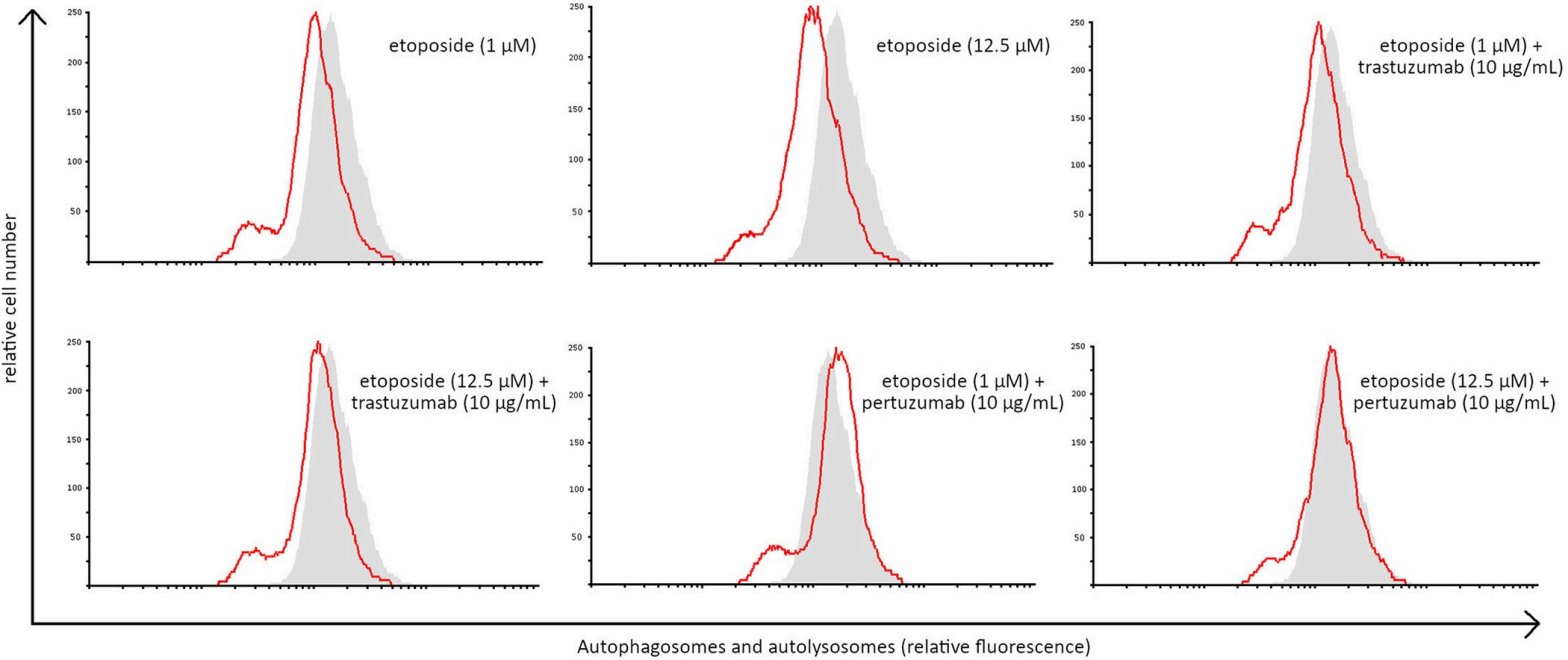
Autophagy induction in AGS gastric cancer cells measured by flow cytometry using autophagy probe after 24 hours incubation with the tested compounds. Representative histograms were derived from flow cytometric analysis of 10000 cells and show control cells (light grey histogram) and cells concomitantly treated with agents alone and in combination with anti-HER2 monoclonal antibodies (red line).

At the next stage of the research we conducted an autophagy assay to measure the number of autophagosomes and autolysosomes after treatment with etoposide alone and in combination with anti-HER2 monoclonal antibodies. The analysis revealed that autophagy was not induced after 24 hours of incubation. We didn’t observe the higher number of autophagosomes and autolizosomes in comparison with control (Fig 10).

### Combination of etoposide with anti-HER2 monoclonal antibodies decreases the concentration of Beclin-1 in comparison with the untreated control

The Beclin-1 protein is commonly known regulator of autophagy and its decreased level is thought to inhibit that process [18]. The concentration of Beclin-1 in the untreated control was 10 ng/ml. The etoposide and combination of etoposide with anti-HER2 antibodies decreased the concentration of the analyzed protein (p<0.05). The effect was dependent on the dose of etoposide. After 24-hour incubation with the lower dose of etoposide, the concentration was 4.8 ng/ml, whereas after the exposition of cells to the higher dose of the chemotherapeutic agent, the concentration decreased to 2.8 ng/ml.

The combination of etoposide and trastuzumab more efficiently decreased the concentration of Beclin-1 to 6.28 ng/ml after treatment with the lower dose of etoposide (1 μM) and 3.32 ng/ml after the incubation of cells with the higher dose of topoisomerase II inhibitor (12.5 μM).

**Fig 11:**
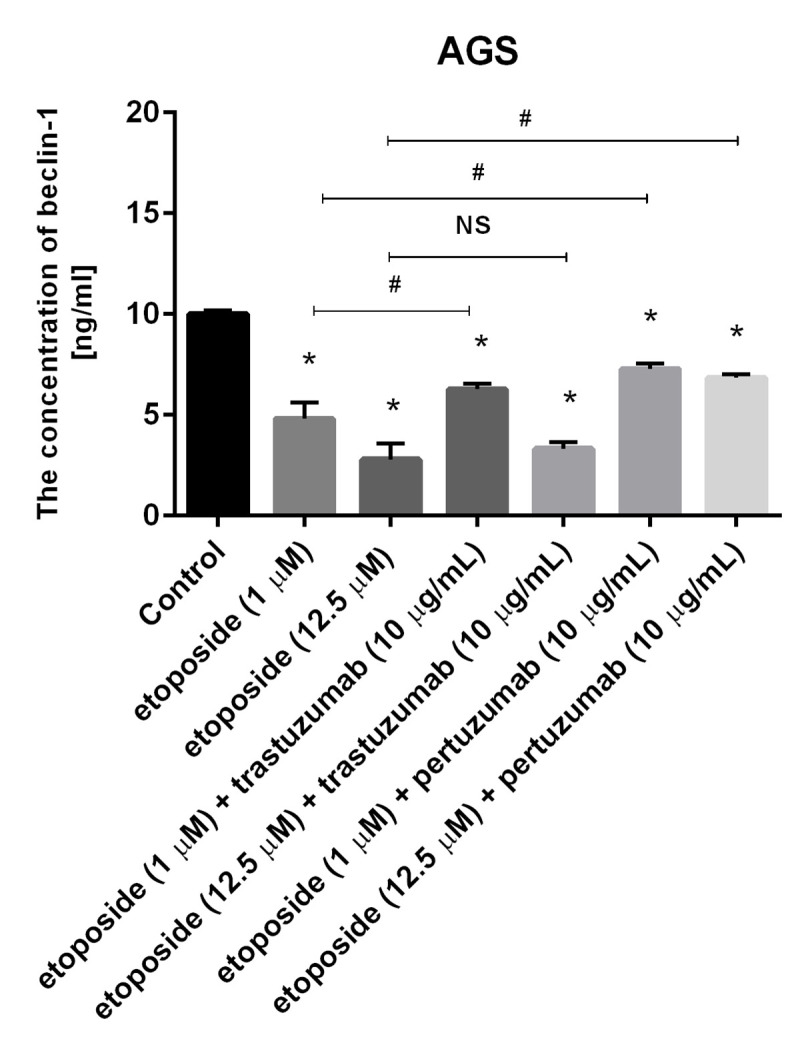
Concentration of Beclin-1 in human gastric cancer AGS cells after 24h incubation with etoposide alone and its combination with trastuzumab or pertuzumab (trastuzumab + etoposide, pertuzumab + etoposide). Data presented in ng/mL. The ANOVA, and Tukey tests were used to demonstrate differences between the control cells and the cells exposed to varying concentrations of the tested compounds. *P<0.05 vs. control group; #p<0.05. Ns, not significant.

The combination of etoposide and pertuzumab was not as good at decreasing the level of Beclin- 1 as etoposide and trastuzumab, but the concentration was lower than in the control. We detected 7.27 ng/ml of the tested protein after incubation with etoposide (1 μM) and pertuzumab. The increase of the dose of etoposide to 12.5 μM led to a decrease of Beclin-1 concentration to 6.85 ng/ml. All values were statistically significant in comparison with the control (p< 0.05) (Fig 11).

### Combination of etoposide with anti-HER2 monoclonal antibodies decreases the concentration of microtubule-associated protein 1 light chain 3A and 3B (LC3A and LC3B)

**Fig 12:**
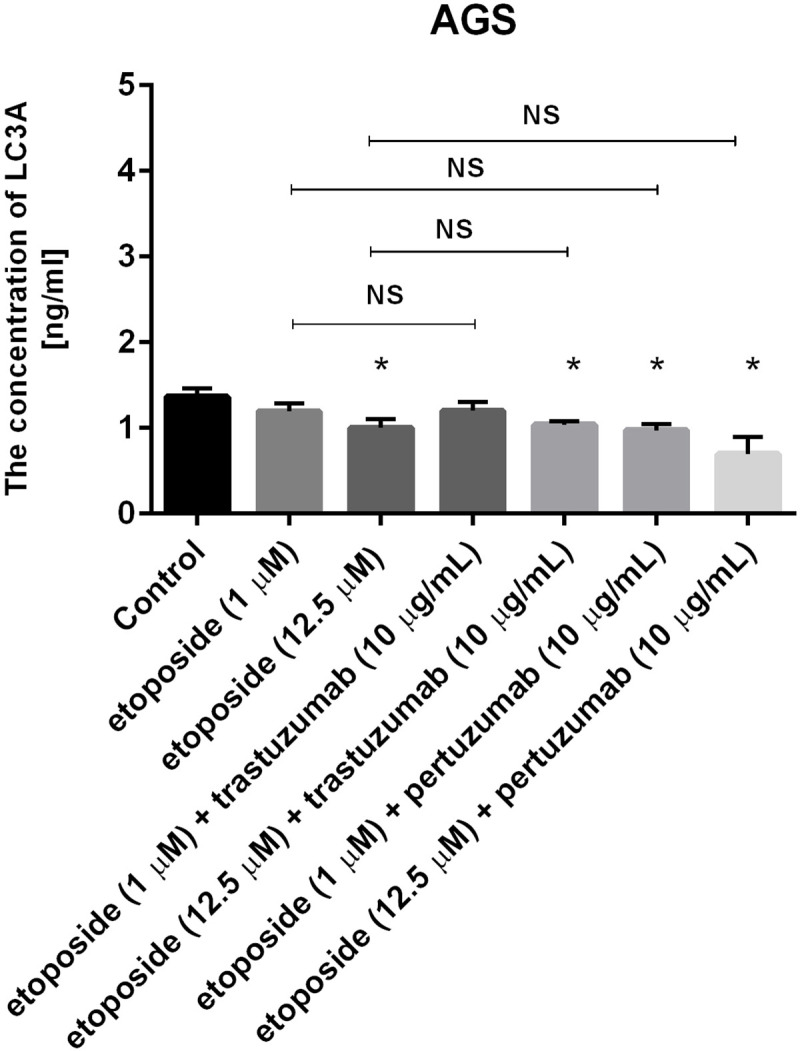
Concentration of LC3A in human gastric cancer AGS cells after 24h incubation with etoposide alone and its combination with trastuzumab or pertuzumab (trastuzumab + etoposide, pertuzumab + etoposide). Data presented in ng/mL. The ANOVA, and Tukey tests were used to demonstrate differences between the control cells and the cells exposed to varying concentrations of the tested compounds. *P<0.05 vs. control group; #p<0.05. Ns, not significant.

**Fig 13:**
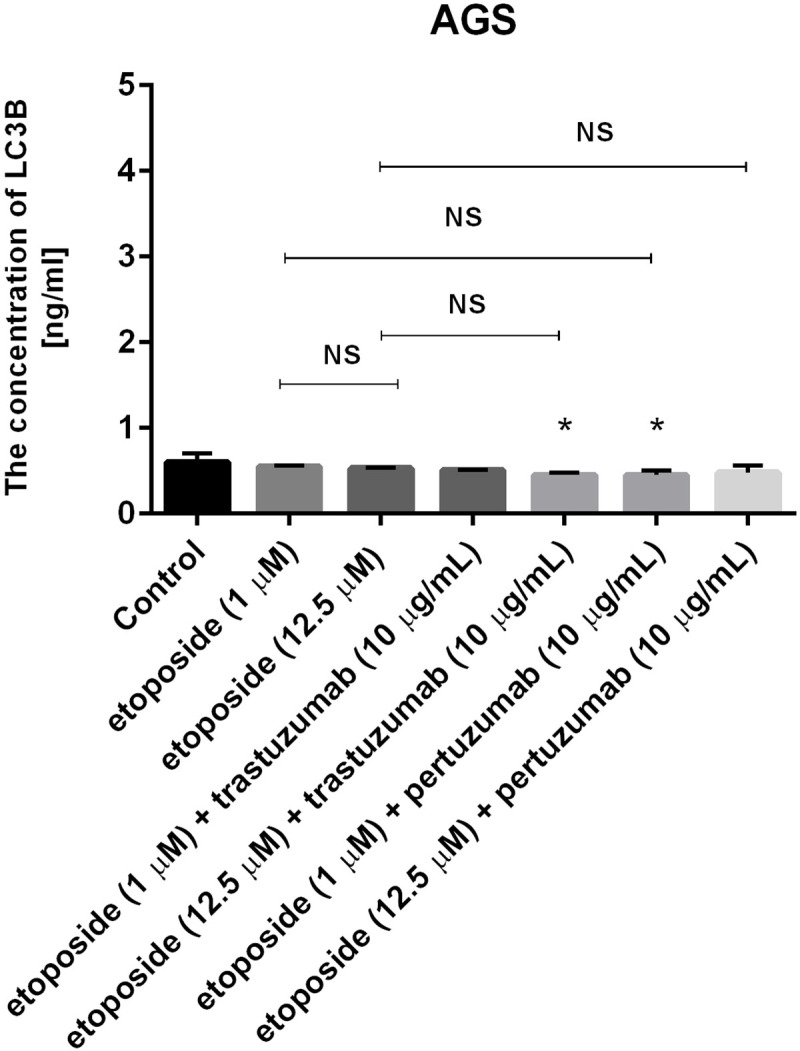
Concentration of LC3B in human gastric cancer AGS cells after 24h incubation with etoposide alone and its combination with trastuzumab or pertuzumab (trastuzumab + etoposide, pertuzumab + etoposide). Data presented in ng/mL. The ANOVA, and Tukey tests were used to demonstrate differences between the control cells and the cells exposed to varying concentrations of the tested compounds. *P<0.05 vs. control group; #p<0.05. Ns, not significant.

**Fig 14:**
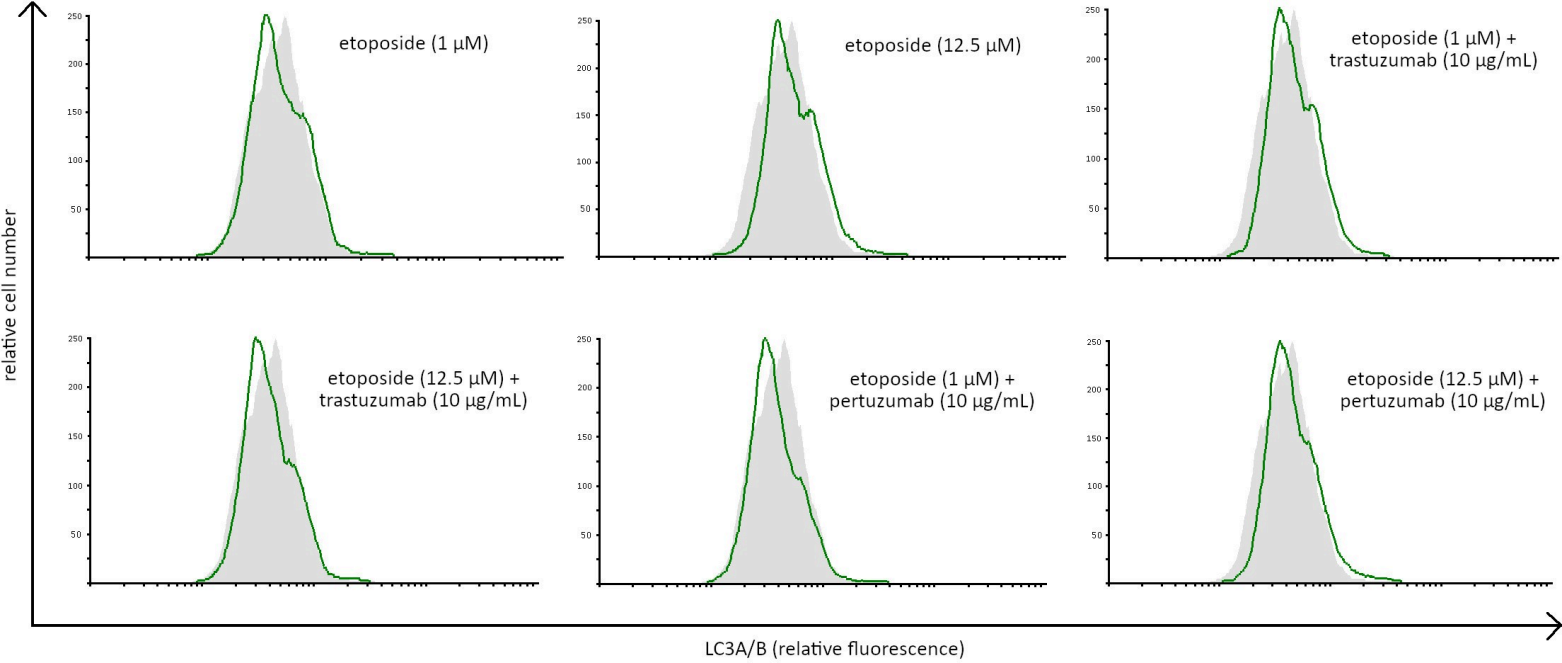
Expression of LC3A/B in AGS gastric cancer cells exposed to the tested compounds. Representative histograms were derived from flow cytometric analysis of 10000 cells and show control cells (light grey histogram) and cells concomitantly treated with agents alone and in combination with anti-HER2 monoclonal antibodies (green line).

The concentrations of LC3A and LC3B were assessed to confirm that autophagy was not induced after 24-hour incubation with the tested agents. All the studied compounds decreased the levels of LC3A and LC3B after 24 hours of incubation in comparison with the untreated cells (Figs 12 and 13). To confirm the results obtained by ELISA technique, the expression of LC3A/B was analyzed in AGS gastric cancer cells by flow cytometry. We didn’t observe higher LC3A/B expression in comparison with untreated control (Fig 14).

## Discussion

Gastric cancer (GC) is a multifactorial disease and remains a leading cause of death worldwide [19]. Better knowledge about the pathogenesis of gastric cancer, especially about the dysfunction of molecular signaling pathways, led to the selection of molecular targets that play a significant role in the development and progression of the disease. Numerous classes of drugs belonging to HER2, vascular endothelial growth factor/vascular endothelial growth factor receptor (VEGF/VEGFR), epidermal growth factor receptor (EGFR), fibroblast growth factor (FGF), phosphoinositide 3-kinase/mammalian target of rapamycin (PI3K/mTOR), and poly-ADP-ribose polymerase (PARP) inhibitors are under investigation in preclinical studies and clinical trials and promising results have been demonstrated [5,20–24]. Some of the drugs (trastuzumab, ramucirumab, apatinib) were accepted by the FDA in the treatment of GC [1]. The experience of our research group proved that the combination of anti-MUC1 monoclonal antibody with cisplatin or etoposide led to a better anticancer effect in breast cancer cells than monotherapy [25,26]. Additionally, a combination of natural compounds (*Nigella sativa* seed oil or extract) with etoposide also enhanced the proapoptotic activity in gastric cancer cells [27]. The published data demonstrated that the anticancer strategy focused on specific molecular target, which is overexpressed in cancer cells is a very promising way of treatment. Overexpression of HER2 in gastric cancer is associated with poor prognosis for patients and the therapy based on looking for novel anticancer agents and strategies are leading and priority the direction of research studies [28].

Several studies have demonstrated the benefits from combining trastuzumab with conventional chemotherapeutic agents such as: doxorubicin, paclitaxel, cisplatin, carboplatin, vinorelbine and taxanes [29–36], but there are no studies where the interaction between trastuzumab or pertuzumab with etoposide is described. Fujimoto-Ouchi K. et al. showed that the combination consisting of: cisplatin, capecitabine and trastuzumab significantly inhibited tumor growth in HER2 positive gastric cancer models [37]. The results from the ToGA trial demonstrated that combination of anti-HER2 antibody (trastuzumab) with capecitabine and cisplatin, or with fluorouracil plus cisplatin led to an increase in overall survival of patients with HER2-positive advanced gastric cancer or gastroesophageal cancer in comparison with chemotherapeutic agents alone [5,38]. The other research demonstrated that trastuzumab used together with chemotherapy (epirubicin and cyclophosphamide) increased overall survival (OS) as well as improved the effectiveness of therapy and reduced the duration of treatment [39]. Patients with HER-2 positive locally advanced oesophago-gastric adenocarcinoma received chemotherapy, trastuzumab and chemoradiotherapy in the TOXAG study [40]. Additionally, trastuzumab plus (capecitabine/oxaliplatin) is also tested in advanced gastric cancer [41].

The anticancer activity of pertuzumab in combination with trastuzumab and chemotherapy was analyzed in the JOSHUA trial and the findings were the basis for the JACOB study (NCT01774786). The final results from the JACOB clinical trial were published, where the combination of trastuzumab, pertuzumab and chemotherapy (cisplatin, capacetabine or 5-fluorouracil) was tested in patients with HER2-positive metastatic gastric or gastro-oesophageal junction cancer. Unfortunately, the observations did not demonstrate an improvement in overall patient survival. There was no significant difference between combinations consisting of dual anti-HER2 antibodies: pertuzumab, trastuzumab and chemotherapy in comparison with trastuzumab and chemotherapy [42,43]. The conclusion from this study is that treatment with dual anti-HER2 antibodies did not increase the efficacy of therapy, thus other chemotherapeutic agents should be taken into account or new derivatives of drugs should be synthesized.

Our study proved that breast cancer cells were not susceptible to etoposide alone and its combination with anti-HER2 antibodies. The promising results were obtained from the experiments performed in AGS gastric cancer cells. In our study, we checked the cytotoxic and antiproliferative effect of etoposide alone tested in two doses (1 and 12.5 μM) and in combination with trastuzumab or pertuzumab in AGS gastric cancer cells. We proved that etoposide (12.5 μM) with pertuzumab possessed the most significant anticancer activity. Pertuzumab and etoposide inhibited viability and proliferation more effectively than single treatment or a combination consisting of trastuzumab and etoposide. Apoptosis represents a promising target in anticancer therapy. Deregulation of programmed cell death is associated with uncontrolled cell proliferation, progression of cancer as well as cancer resistance to therapeutic agents. The programmed cell death may go through extrinsic pathway, which begins when a death ligand (Fas-L, TRAIL, TNF) binds to tumor necrosis factor (TNF) family death receptors. An adaptor protein binds to the receptor and DISC (death-inducing signaling complex) is formed. It consists of the adaptor protein and procaspases-8 and -10. Active caspase-8 leads to activation of the executioner caspases such as caspases-3, -6 and -7 and BID, whose activation leads to the cell death. Mitochondria and mitochondrial proteins play an important role in intrinsic apoptotic pathway. The most important events of intrinsic pathway include mitochondrial outer membrane permeabilization, the release of intermembrane proteins like cytochrome c and activation of caspase-9, which is responsible for activation of the executioner caspases-3 and -7. Their action leads to cell death [44]. Pertuzumab with etoposide led to the induction of apoptosis, where a high number of cells with active caspase-8 and caspase-9 as well as decreased MMP were demonstrated. A major form of regulated cell death is necroptosis, which play a pivotal role in oncogenesis and cancer metastasis [45]. This process is controlled by receptor-interacting protein (RIP) kinases RIPK1, RIPK3 and MLKL. It was discovered that this mode of cell death was activated in caspase-8 deficient colorectal cancer and led to the significant tumor regression in mice in response to Smac mimetic [46]. The results support the data that activation of necroptosis is a mechanism to overcome apoptosis resistance. Autophagy is a process of self-degradation and is classified into macroautophagy, selective autophagy, microautophagy and chaperone-mediated autophagy (CMA). There are potential pathways of the relationship between apoptosis and autophagy and one of them is autophagy suppression and induction of apoptosis, which was presented in this paper. p53, Bcl-2/Beclin 1, Atg proteins, p62 or caspases are the key factors connecting apoptosis and autophagy. As it was mentioned above caspases are engaged in both extrinsic and intrinsic apoptotic pathways, but also affect the autophagy process [47]. Oral et al. demonstrated that overexpression of caspase-8 promotes the degradation of Atg3 protein and finally inhibits its pro-autophagic activity [48]. Some autophagy inhibiotors undergo preclinical and clinical trials. Hydroxychloroquine, verteporfin, and clarithromycin are still under clinical investigations [NCT04524702, NCT03774472, NCT03067051, NCT03033225, NCT04302324, NCT04063189, NCT02542657, NCT03031483, NCT00461084]. 3-Methyladenine, Lys05, 7-methyl-5-phenylpyrazolo[4,3-*e*]tetrazolo[4,5-*b*][1,2,4]triazine sulfonamide derivatives, miconazole and α –Hederin deserve for attention in a group of autophagy inhibitors undergoing preclinical trials [49–53]. In our study, we checked the concentrations of important proteins engaged in autophagy and we proved that Beclin-1 concentration was dependent on the chemotherapeutic agent dose. The higher dose of etoposide in combination with trastuzumab or pertuzumab led to a lower concentration of the analyzed protein in cell lysates. All the studied compounds decreased the levels of LC3A and LC3B after 24 hours of incubation in comparison with the untreated cells. Flow cytometric analysis confirmed that autophagy was not induced after treatment with the monotherapy as well as combination strategy. Our data supports the thesis that the inhibition of autophagy increases the susceptibility of cancer cells to the treatment and favors apoptosis [54].

### Conclusions

The combination of pertuzumab with etoposide represents a promising strategy aimed at inhibition of viability and proliferation of gastric cancer cells. The mechanism of action includes induction of both extrinsic and intrinsic apoptotic pathway, where increased activity of caspase-8 and caspase-9 was demonstrated. Additionally, the analyzed combination did not induce autophagy. The combination of anti-HER2 antibody with etoposide prevent the autophagosomes and autolizosomes formation and did not increase the concentrations of Beclin-1, LC3A and LC3B in comparison with untreated control. We proved that such a dual treatment regimen allowed to achieve relevant efficacy, but further studies are required.
